# Application scope of knowledge graphs in nursing: a scoping review

**DOI:** 10.3389/fpubh.2026.1763063

**Published:** 2026-04-15

**Authors:** Yu Wang, Xue Pan, Hanghang Jin, Rongyue Guo, Kechen Zhu, Wanying Zhao, Xin Wang

**Affiliations:** School of Nursing and Health, Zhengzhou University, Zhengzhou, China

**Keywords:** artificial intelligence, knowledge graphs, nursing, nursing informatics, scoping review

## Abstract

**Objective:**

The application of knowledge graph technology in the healthcare field is increasingly in-depth, yet there is a lack of literature that systematically sorts out the overall research landscape of its application in nursing from a macro perspective. This study aimed to systematically depict the research panorama of knowledge graph applications in nursing, stratify existing studies by stages through the construction of an exploratory evidence maturity framework, reveal structural gaps and translation barriers, and provide insights for subsequent in-depth research and practical applications.

**Methods:**

This study adopted the Arksey scoping review reporting framework and followed the PRISMA-ScR checklist for reporting. We systematically searched databases including Wanfang, CNKI, VIP, SinoMed, PubMed, Embase, Web of Science, CINAHL, and the Cochrane Library, and summarized and analyzed the included articles. The research results were comprehensively collated and divided into five phases: the Construction Phase, System Performance Evaluation Phase, Usability Evaluation Phase, Preliminary Application Phase, and Application Phase.

**Results:**

A total of 30 studies were included, with methodological studies as the main research design (*n* = 15), covering themes such as nursing education, disease management, health education, clinical nursing decision support, risk prediction, and psychological support. Analysis based on the evidence maturity framework showed that there were 12 studies (40%) in the knowledge graph Construction Phase, six (20%) in the System Performance Evaluation Phase, eight (26.7%) in the Usability Evaluation Phase, one (3.3%) in the Preliminary Application Phase, and three (10%) in the Application Phase. The research focus exhibited obvious domain bias: studies on nursing education scenarios dominated, while those on clinical nursing scenarios were relatively scarce.

**Conclusion:**

Knowledge graph research in nursing is still in the exploratory stage, dominated by evidence on technical construction, with no objective validation of its clinical application effects. Future research should adopt an evidence-driven approach, focusing on clinical application, optimizing study design and advancing data standardization, thereby enabling knowledge graphs to deliver evidence-based support in nursing practice.

**Scoping review registration:**

https://osf.io/, identifier 10.17605/OSF.IO/F7SB5.

## 1 Introduction

Over the past decade, the nursing discipline has experienced exponential growth driven by evidence-based practice, precision nursing, and artificial intelligence. PubMed alone has indexed more than 180,000 nursing articles from 2020 to 2024, and the update cycle of clinical guidelines has shortened to ≤18 months (1). However, knowledge is scattered as free text in electronic medical records, nursing documentation, clinical guidelines, and educational databases, forming severe information silos. Traditional reviews and expert consensus fail to integrate multi-source evidence in real time, resulting in clinical nurses spending more than 30 min searching for the best practice for bedside decision-making (2). With the rapid development of cutting-edge technologies such as big data and artificial intelligence, the in-depth integration of the healthcare field with information technology is driving nursing into a critical stage of intelligent upgrading. The International Council of Nurses (ICN) Strategic Plan (2021–2025) calls on all countries to leverage big data, artificial intelligence, and mobile technologies to seamlessly connect nursing documentation, clinical decision-making, and patient self-management, so as to realize smart nursing and precision nursing (3).

As an important subfield of artificial intelligence, knowledge graph technology has ushered in new development opportunities in recent years. On the one hand, the volume of healthcare data has exploded, with electronic medical record systems, medical Internet of Things devices, and other tools accumulating massive amounts of clinical nursing data; on the other hand, the continuous maturity of technologies such as natural language processing and machine learning has provided strong support for the construction, population, and reasoning of knowledge graphs, enabling their application in the medical field to be gradually deepened and expanded (4). Knowledge graphs have been proven to improve the interpretability and accuracy of models in scenarios such as drug repurposing and disease risk prediction (5). In the healthcare industry, ensuring traceable results and reliable and credible data sources is crucial. Compared with the black-box mechanism of many current large language models with flawed output quality (6), the white-box mechanism of knowledge graphs can explain the generated results. The traceability and real data sources of knowledge graphs can be used to build more excellent medical artificial intelligence systems (7).

Despite the increasing use of knowledge graphs in healthcare, the term itself is often used interchangeably with related but distinct concepts such as ontologies and semantic networks. In this review, we adopt a precise operational definition: a knowledge graph is a structured knowledge representation that uses entities, relationships, and attributes to form a network, enabling semantic query and reasoning. This distinguishes it from ontologies, which define formal vocabularies and hierarchical relationships but typically lack instance-level integration and dynamic querying capabilities, and from semantic networks, which are less formalized and lack standardized reasoning mechanisms. Clarifying this boundary is essential for interpreting the scope and contribution of the studies included in this review.

Research on the application of knowledge graphs in nursing is still in the stage of continuous exploration and improvement. Although some review studies have been published, most focus on specific application fields such as dietary management for patients with chronic diseases (8) and rare diseases (9), and the total number is limited. There is still a lack of literature that systematically sorts out the overall research landscape, development stages, and methodological gaps of knowledge graphs in nursing from a macro perspective. Although existing reviews have summarized partial progress, they fail to present the overall layout and evolutionary context, nor to distinguish and stratify the maturity of evidence at different levels. The lack of this holistic perspective may make it difficult for current research to locate its position in the overall landscape and also hinder the identification of a clear path from proof-of-concept to practical translation. There is still a lack of a comprehensive portrait of how far nursing knowledge graph research has progressed, which themes it covers, and what methodological gaps exist (10), making it necessary to conduct an overall systematic sorting. In view of the wide range of current research types and diverse research methods, scoping reviews help sort out various types of evidence, clarify conceptual boundaries, and identify knowledge gaps in emerging fields. Accordingly, this study adopted a scoping review method (11) to systematically depict the research panorama of knowledge graph applications in nursing, stratify existing studies by stages through the construction of an exploratory evidence maturity framework, reveal structural gaps and translation barriers at different current levels, and provide insights for subsequent in-depth research and practical applications.

## 2 Methods

This study adopted the Arksey scoping review reporting framework (11), which includes the following steps: (i) stating the research objective and research questions; (ii) developing a search strategy; (iii) defining inclusion and exclusion criteria for literature screening and data extraction; (iv) conducting statistical analysis; (v) summarizing research results; (vi) identifying deficiencies in the research field and future research directions. The study followed the PRISMA Extension for Scoping Reviews (PRISMA-ScR) checklist for reporting (12) (see Supplementary Appendix S1 for details). The protocol of this review has been registered in OSF (DOI: 10.17605/OSF.IO/F7SB5).

### 2.1 Definition of research questions

Drawing on the phased progressive logic of the NIH Stage Model for Behavioral Intervention Development proposed by the U.S. National Institutes of Health (13), this study made adaptive adjustments according to the characteristics of the included studies and developed an evidence maturity framework to organize the research results. This review aimed to: (1) systematically describe the overall landscape of knowledge graph applications in nursing; (2) summarize the methodological characteristics, health themes, implementation methods, and reported outcomes of knowledge graphs in phased stratification; (3) reveal structural fractures and translation barriers in the currently included studies, and provide insights for subsequent in-depth research and practical applications.

### 2.2 Search strategy

We systematically searched the Wanfang Database, China National Knowledge Infrastructure (CNKI), VIP Database, SinoMed, PubMed, Embase, Web of Science, CINAHL, and the Cochrane Library. A combination of subject headings and free words was used for the search, with the search time frame from the establishment of each database to August 2025. The primary search included terms related to “Knowledge Graphs” and “semantic network” and “ontology” and “ontology” and “Nursing” and “Nursing Care.” In addition, the references of relevant literatures were manually retrieved to ensure the comprehensiveness of the included literatures (see Supplementary Appendix S2 for the complete search strategy).

### 2.3 Literature inclusion and exclusion criteria

Based on the classic Population, Concept, and Context (PCC) framework, this study systematically formulated literature inclusion and exclusion criteria; combined with research needs, the category of Study Design was added to the original dimensions of the PCC framework to optimize the scientificity and pertinence of screening, as shown in Table 1.

**Table 1:** Literature inclusion and exclusion criteria.

Category	Inclusion criteria	Exclusion criteria
Population	Nursing students, nurses, or patients/caregivers in nursing-related contexts	① Patients/caregivers who only received medical treatment without nursing-related care and intervention; ② Healthy subjects without any nursing care needs
Concept	Studies involving the application of knowledge graphs (KG) in nursing, including at least one identifiable KG feature: (i) explicit entity-relationship representation; (ii) triple/RDF or graph database implementation; (iii) knowledge query and/or reasoning on graph structures; (iv) KG-based system output (e.g., recommendation, Q&A, prediction)	Studies focusing only on the technical principles of knowledge graphs or theoretical discussions
Context	Nursing education, disease management, risk prediction, health education, clinical nursing decision support, psychological support, and other nursing-related research content	Healthcare scenarios outside the core nursing field (e.g., pure clinical medicine, pharmacy, public health, etc.)
Study design	Major empirical studies (construction, evaluation, or application); randomized controlled trials, quasi-experiments, cohort studies, cross-sectional studies, qualitative studies	Study protocols without results; For duplicate publications, overlapping data, or multiple articles focusing on different dimensions of the same research content, only the most informative one was retained, and the others were excluded

### 2.4 Literature screening and data extraction

After deduplication using EndNote, two evidence-based trained authors (W.Y. and Z.K.C.) screened the titles and abstracts according to the above criteria. Disagreements were resolved through discussion with a third author (J.H.H.). The full texts of eligible literatures were retrieved and evaluated for inclusion by two authors (W.Y. and G.R.Y.), and all disagreements were resolved through discussion among four authors (W.Y., J.H.H., Z.K.C., and G.R.Y.). The two researchers had a high degree of consistency in the title and abstract screening and full-text screening stages; disagreements were mainly concentrated in the definition of research stages and classification of outcome types, which have been resolved through discussion. The screening process was presented using a PRISMA flow diagram to clearly show the screening results of each step. The design of the data extraction form in this study adopted an iterative team calibration strategy: first, based on the research questions and preliminary literature reading, the research team discussed and determined the initial extraction fields covering bibliometric characteristics (author, year, country), methodological attributes (study design, data source, analysis method), contextual characteristics (research scenario, population characteristics), and main research findings. The field definitions were revised and coding options were added or deleted through pre-experiments, and a standardized extraction tool was finally formed. The extracted data included author, year, country, data source, study type, health theme, knowledge graph carrier type, and outcome indicators. To ensure traceability of the classification, the following variables were operationally defined: data sources were coded using an alphanumeric system (see footnotes of Table 2); health theme referred to the nursing scenario in which the knowledge graph was applied, categorized into nursing education, disease management, health education, clinical nursing decision support, risk prediction, and psychological support; carrier type indicated the final delivery format of the knowledge graph, including visualization tools, Q&A systems, prediction models, teaching platforms, mHealth applications, and nursing information systems; outcome indicators were grouped into four categories: knowledge graph scale metrics, technical performance metrics, subjective perception metrics, and objective effect metrics; evidence maturity phase was classified according to the five-phase framework established in Section 2.5. Two authors (W.Y. and P.X.) extracted and summarized the information of the included literatures according to the preset data extraction form, and the extracted data were checked for accuracy by two authors (Z.W.Y. and W.X.).

**Table 2:** Basic characteristics of the included literatures.

First author	Year	Country	Research object	Data source	Study type	Health theme	Carrier type	Outcome indicators	Evidence maturity phase
Li et al. (14)	2023	China	Patients with diabetes	A	Quantitative (retrospective cohort study)	Risk prediction	Prediction model	Accuracy and discrimination of the prediction model	System performance evaluation
Li et al. (15)	2020	USA	Patients with Alzheimer’s disease	B, E	Quantitative (single-group pre–post test)	Disease management	mHealth application	Conversation success rate, correctness, and qualitative feedback	Usability evaluation
Liu et al. (16)	2025	China	Undergraduate nursing students	E, D	Qualitative study	Nursing education	Visualization tool	Students’ perceived usefulness, usability feedback and improvement suggestions of KGs	Usability evaluation
Qiao et al. (17)	2024	China	Patients with AD-asthma	E, G	Methodological study	Disease management	Visualization tool	Knowledge graph scale indicators	Construction
Leng et al. (18)	2023	China	Patients with dementia	H, E	Mixed-methods research	Disease management	Mobile application	Usability and satisfaction	Usability evaluation
Zhang et al. (19)	2022	Australia	Patients with dementia	A, F	Methodological study	Health education	Visualization tool	Knowledge graph scale indicators	Construction
Ding et al. (20)	2024	China	Breast cancer patients with CIF	E	Methodological study	Disease management	Visualization tool	Knowledge graph scale indicators	Construction
Wang et al. (21)	2022	China	Patients with KOA	E, G	Methodological study	Health education	Visualization tool	Knowledge graph scale indicators	Construction
Xu et al. (22)	2024	China	Patients with heart failure	E, G	Methodological study	Clinical nursing decision support	Visualization tool	Knowledge graph scale indicators	Construction
Li et al. (23)	2024	China	Elderly patients with chronic diseases	D, E, H	Qualitative study	Disease management	Nursing information system	Content validity of system output and perceived usefulness	Usability evaluation
Qin et al. (24)	2025	China	Patients with diabetes	F	Methodological study	Disease management	Q&A system	Knowledge graph scale indicators	Construction
Carvalho et al. (25)	2023	Portugal	ICU patients	B	Quantitative (retrospective cohort study)	Risk prediction	Prediction model	Accuracy and discrimination of the prediction model	System performance evaluation
Papadakis et al. (26)	2023	UK	Patients with ADHD	A, E	Methodological study	Clinical nursing decision support	Visualization tool	Correctness and relevance	System performance evaluation
Wang et al. (27)	2020	China	Patients with type 2 diabetes	E	Methodological study	Risk prediction	Prediction model	Knowledge graph scale indicators, accuracy of risk score calculation	System performance evaluation
Kang et al. (28)	2024	China	Elderly population	D, E	Methodological study	Health education	Nursing information system	Knowledge graph scale indicators	Construction
Wang et al. (29)	2024	USA	Cognitively intact older adults	G	Mixed-methods research	Psychological support	mHealth application	User satisfaction and effectiveness; usage experience, advantages/ disadvantages and improvement suggestions	Usability evaluation
Sarnai et al. (30)	2024	USA	Patients with diabetes	A, C, G	Methodological study	Disease management	Nursing information system	Accuracy of the prediction model	System performance evaluation
Patterson et al. (31)	2024	USA	Patients with multiple sclerosis	A	Quantitative (retrospective cohort study)	Risk prediction	Prediction model	Accuracy and discrimination of the prediction model	System performance evaluation
Singla et al. (32)	2024	Canada	Patients with neurodevelopmental disorders	B	Mixed-methods research	Disease management	mHealth application	Resource relevance score, usability and feasibility, query response time	Usability evaluation
Zhang et al. (33)	2024	China	Nursing students	H	Methodological study	Nursing education	Visualization tool	Knowledge graph scale indicators	Construction
Zhang et al. (34)	2024	China	Nursing students	H	Quantitative (single-group descriptive)	Nursing education	Teaching platform	Quality of life and work, perceived usefulness	Usability evaluation
Zhang et al. (35)	2025	China	Nursing students	H	Quantitative (single-group descriptive)	Nursing education	Teaching platform	Usability and user experience	Usability evaluation
Xiong et al. (36)	2023	China	General patients	B, H	Methodological study	Clinical nursing decision support	Nursing information system	Knowledge graph scale indicators	Construction
Chen et al. (37)	2024	China	Nursing students	F, H	Methodological study	Nursing education	Visualization tool	Knowledge graph scale indicators	Construction
Rong et al. (38)	2024	China	Nursing students	H	Mixed-methods research	Nursing education	Teaching platform	Independent learning ability, teaching effect, perception and experience, knowledge point coverage	Application
Yang et al. (39)	2024	China	Nursing students	E, F, H	Mixed-methods research	Nursing education	Teaching platform	Learning duration, practice times, satisfaction and feelings	Preliminary application
Bao et al. (40)	2024	China	Nursing students	H	Quantitative (non-randomized controlled trial)	Nursing education	Teaching platform	Total course scores, online learning readiness; knowledge point completion/mastery rate, platform visit volume	Application
Song et al. (41)	2024	China	Nursing students	H	Quantitative (historical controlled trial)	Nursing education	Teaching platform	Cross-grade theoretical assessment scores, satisfaction percentage, mastery compliance rate, number of knowledge themes, types and quantity of resources	Application
Chen et al. (42)	2024	China	Patients with enterostomy	D, F, H	Methodological study	Disease management	Q&A system	Knowledge graph scale indicators	Construction
Chen et al. (43)	2024	China	Nursing staff	D, H	Methodological study	Nursing education	Q&A system	Knowledge graph scale indicators	Construction

Data sources: A, Electronic health records; B, Clinical databases; C, Smart device data; D, Authoritative knowledge data (including clinical guidelines, expert consensus, evidence summaries); E, Academic literatures; F, Healthcare websites; G, Personal survey data; H, Teaching-related materials (including textbooks, syllabi, auxiliary materials); AD, Atopic dermatitis; CIF, Cancer-related fatigue; KOA, Knee osteoarthritis; ADHD, Attention deficit hyperactivity disorder.

### 2.5 Data analysis and synthesis

Two different methods were adopted for data analysis: (1) Descriptive analysis: Summarizing the research characteristics (e.g., publication year, country, research method) to understand the scope and distribution of the literature; (2) Content analysis based on the exploratory framework constructed by the research team: To better explain the evidence strength in heterogeneous studies, the research team discussed and constructed an exploratory evidence maturity framework for knowledge graph applications in nursing. This framework drew on the phased progressive logic of the NIH Stage Model for Behavioral Intervention Development (13) with adaptive adjustments. The division of “phases” in the framework was based on observable characteristics such as whether real users were included, whether controls were set, and whether objective outcomes were measured, rather than a value judgment on the quality of research design. The studies were divided into five phases:

- Construction Phase: Focusing only on ontology design, entity/relationship extraction, and data fusion of the knowledge graph, with no subsequent evaluation or application links;
- System Performance Evaluation Phase: Conducting testing and verification of the knowledge graph in a laboratory environment only after its construction, with no user use or clinical application;
- Usability Evaluation Phase: Conducting evaluation in a laboratory environment or the corresponding research scenario, but focusing on user experience (usability, scenario adaptability), with no hard clinical outcomes or technical performance evaluation;
- Preliminary Application Phase: Implementing pilot research on knowledge graphs in the corresponding research scenario, adopting before-and-after comparison or single-group design, measuring the preliminary participation of research objects or descriptive outcomes, with no outcome improvement;
- Application Phase: Applying knowledge graphs in specific scenarios with a controlled comparison design and improved outcomes of research objects, with indicators focusing on outcome-oriented and objective outcomes.

### 2.6 Critical appraisal

In accordance with the JBI guidelines for scoping reviews, no formal critical appraisal was conducted. The purpose of this review was to sort out the scope and characteristics of evidence on knowledge graphs in nursing, rather than to evaluate the validity or quality of the studies. The included studies varied significantly in design, results, and methodological rigor, making them unsuitable for standardized quality comparison. In addition, most studies were methodological, reflecting that this field is still in the initial stage with limited empirical evidence.

## 3 Results

**Figure 1:**
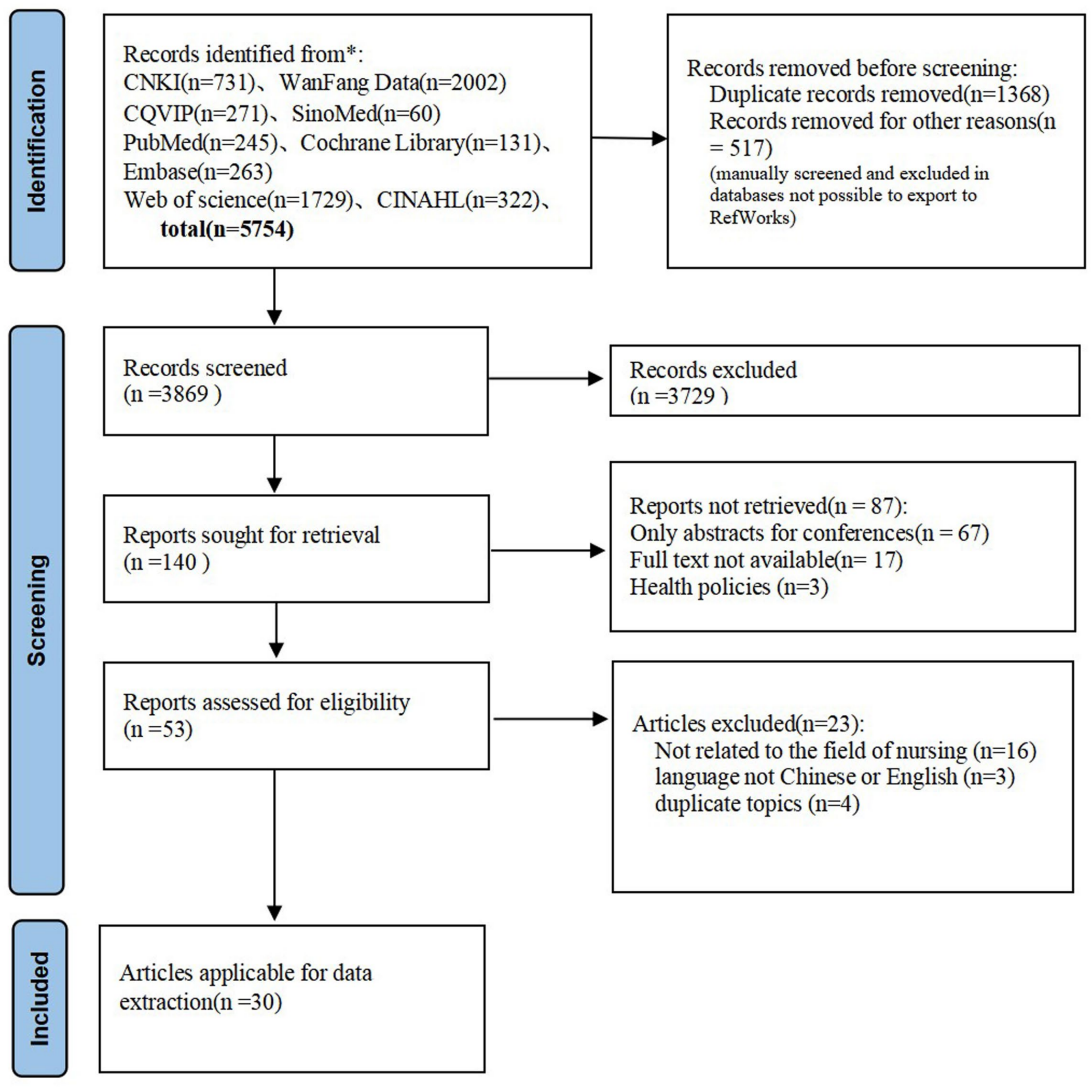
Literature screening process.

A total of 5,754 literatures were retrieved from the databases. After removing duplicate literatures using EndNote software, 3,869 remained. A total of 140 literatures were retained after reading the titles and abstracts, and finally 30 studies (14–43) meeting the inclusion criteria were included after full-text reading (shown in Figure 1, PRISMA flow diagram).

### 3.1 Basic characteristics of the included literatures

Table 2 presents the basic characteristics of the 30 included studies. The geographical distribution showed a unipolar concentrated pattern: China contributed the vast majority of the studies (*n* = 22) (14, 16–18, 20–24, 27, 28, 33–43), followed by the United States (15, 29–31), with one study each from Australia (19), Portugal (25), the United Kingdom (26), and Canada (32). In terms of temporal distribution, the studies were concentrated in the period from 2020 to 2025, with the largest number published in 2024 (*n* = 18) (17, 20, 22, 23, 28–34, 37–43), forming an obvious temporal clustering effect. The distribution of study design types was dominated by methodological studies (*n* = 15) (17, 19–22, 24, 26–28, 30, 33, 36, 37, 42, 43), mainly focusing on ontology construction; followed by quantitative studies (*n* = 8) (14, 15, 25, 31, 34, 35, 40, 41), including retrospective cohort studies (14, 25, 31), non-randomized controlled trials (40), historical controlled trials (41), single-group descriptive studies (34, 35), and single-group pre-post test studies (15); mixed-methods research (18, 29, 32, 38, 39) and qualitative studies (16, 23) accounted for a relatively low proportion.

### 3.2 Distribution characteristics of studies based on the evidence maturity framework

The 30 studies were distributed across the five evidence maturity phases (Table 3). The specific distribution of each phase in terms of study design, health theme, carrier type, and reported outcomes is detailed in Sections 3.2.1–3.2.5.

**Table 3:** Distribution characteristics of the included literatures by evidence maturity phase.

Evidence maturity phase	Number of studies (*n*)	Distribution of study design types	Sample characteristics	Main outcome types	Key observations
Construction Phase	12 (40%)	Methodological studies (*n* = 12)	Not applicable	Knowledge graph scale indicators^1^	Data sources are dominated by academic literatures and textbooks (9/12), with few studies on real-world data integration. Among the 12 construction-phase studies, 11 involved fully implemented knowledge graphs (including graph databases and query/reasoning functionalities), and one was an ontology-oriented precursor study (Zhang et al. (19), only an OWL ontology, without a graph database or user queries).
System Performance Evaluation Phase	6 (20%)	Methodological studies (*n* = 3); Quantitative studies (*n* = 3)	Laboratory environment, virtual cases	Technical performance indicators^2^	Verification scenarios are laboratory environments or virtual cases with no real patients in clinical settings; technical performance evaluation is not compared with conventional nursing tools.
Usability Evaluation Phase	8 (26.7%)	Quantitative studies (*n* = 3); Qualitative studies (*n* = 2); Mixed-methods research (*n* = 3)	Sample size: 7–56; Convenience sampling	Subjective perception indicators (primary)	No control groups; most studies adopted convenience sampling.
Preliminary Application Phase	1 (3.3%)	Mixed-methods research (*n* = 1)	Sample size: 83; Single group	Subjective perception indicators combined with objective indicators	There was one study at this stage; no changes in patient-related outcomes were reported.
Application Phase	3 (10%)	Quantitative studies (*n* = 2); Mixed-methods research (*n* = 1)	Sample size: 74–298; Non-random grouping	Objective effect indicators (primary) combined with partial subjective perception indicators	Evidence is concentrated in nursing education scenarios; objective outcome indicators are course scores or independent learning ability; follow-up time is limited to the semester, no more than one semester.
Total	30	–	–	–	No randomized controlled trials (RCTs) were found in the study design types; 60% of the evidence is distributed in the Construction and System Performance Evaluation Phases.

^1^Knowledge graph scale indicators: number of entities, types of relationships, number of triples, visualization effect, etc.; ^2^ Technical performance indicators: AUC, RMSE, accuracy, Relevance. etc.

#### 3.2.1 Knowledge graph Construction Phase

A total of 12 studies (17, 19–22, 24, 28, 33, 36, 37, 42, 43) were included in the Construction Phase, with the core task focusing on ontology design, entity/relationship extraction, and data fusion in nursing-related fields. All study designs were methodological with no technical performance verification or real application scenarios involved. Health themes were scattered, covering nursing education (33, 37, 43), disease management (17, 20, 24, 42), health education (19, 21, 28), and clinical nursing decision support (22, 36). The main carrier types of knowledge graphs were visualization tools (17, 19–22, 33, 37), and some studies began to explore embedding in Q&A systems (24, 42, 43). The reported outcome indicators were mainly knowledge graph scale and structural indicators, such as the number of entities, types of relationships, and number of triples.

#### 3.2.2 Knowledge graph System Performance Evaluation Phase

Six studies (14, 25–27, 30, 31) were included in the System Performance Evaluation Phase, all of which verified the technical performance of knowledge graphs in laboratory or offline environments. The study designs included quantitative studies (14, 25, 31) and methodological studies (26, 27, 30), with all quantitative studies being retrospective cohort studies. No real users or real nursing processes were included in these studies. The themes were concentrated on risk prediction (14, 25, 27, 31). The carrier types of knowledge graphs included prediction models (14, 25, 27, 31), nursing information systems (30), and visualization tools (26). The reported outcome indicators in this phase were mainly technical performance parameters, including accuracy indicators of prediction models (e.g., AUC, RMSE) and system operation performance, with some studies supplemented by qualitative descriptions of user experience.

#### 3.2.3 Knowledge graph Usability Evaluation Phase

Eight studies (15, 16, 18, 23, 29, 32, 34, 35) were included in the Usability Evaluation Phase, which began to introduce real users and focused on evaluating the usability and perceived usefulness of knowledge graphs in specific nursing scenarios. The study designs were dominated by mixed-methods research (18, 29, 32) and quantitative studies (15, 34, 35), followed by qualitative studies (16, 23). The sample sizes were generally small, ranging from 7 to 56 participants, and most adopted convenience sampling. The themes included disease management (15, 18, 23, 32), nursing education (16, 34, 35), psychological support (29), and clinical nursing decision support (23). In this phase, knowledge graphs were usually presented in the form of mHealth applications (15, 18, 29, 32), visualization tools (16), nursing information systems (23), and teaching platforms (34, 35). The reported outcome indicators covered subjective evaluation dimensions such as user satisfaction, perceived usability, usage experience, and improvement suggestions, which were mainly obtained through scale evaluation and qualitative interviews.

#### 3.2.4 Knowledge graph Preliminary Application Phase

Only one study (39) was included in the Preliminary Application Phase, which implemented a pilot application of knowledge graphs in specific nursing scenarios. The study design was mixed-methods research, the health theme was nursing education, and the knowledge graph was embedded in a teaching platform for use. The outcome indicators included learning duration, practice times, and satisfaction.

#### 3.2.5 Knowledge graph Application Phase

Three studies (38, 40, 41) were included in the Application Phase, which saw the first appearance of controlled comparison design and improved objective outcomes. All studies were concentrated on the theme of nursing education, with knowledge graphs embedded in teaching platforms. The study designs included non-randomized controlled trials (40), historical controlled trials (41), and mixed-methods research (38). The outcome indicators presented a pattern of three types coexisting: objective outcome indicators included total course scores, cross-grade theoretical assessment scores, and independent learning ability scale scores; subjective outcome indicators included learning readiness and satisfaction; system performance indicators included platform visit volume and knowledge point coverage.

## 4 Discussion

### 4.1 Characteristics of the included studies

Current research on knowledge graphs in nursing is in a developmental stage of rapid expansion and in-depth accumulation. In terms of temporal distribution, the number of studies has risen rapidly from 2022 to 2025, with 15 published in 2024 alone. This expansion in output scale reflects the growing recognition of knowledge graphs as an emerging technical tool. The polarized geographical distribution is also noteworthy: China contributed 22 studies, far exceeding the total of other countries. Although this unipolar concentration reflects China’s high attention to knowledge graphs, it also means that the cross-cultural applicability of the evidence has not been tested, and it remains unknown whether knowledge graphs constructed based on a single national scenario can be transferred to other contexts.

### 4.2 Key findings

From the perspective of evidence maturity, this study systematically sorted out and stratified the application research of knowledge graphs in nursing. Compared with previous reviews on digital health or knowledge graph applications in the healthcare field (8, 9), this study not only focused on the induction of overall research trends and current status, but also emphasized the phase distribution of studies in the evidence generation path and their corresponding outcome characteristics, thus revealing the maturity of the current evidence base and structural gaps in this field. This finding provides a more hierarchical basis for judging the feasibility of further verification, promotion, and policy translation of knowledge graphs in nursing practice. Overall, current research is mainly concentrated in the early stages of knowledge graph construction, technical performance evaluation, and usability evaluation, while the number of studies that have entered real application scenarios and verified application effects with controlled designs is small and highly concentrated in the field of nursing education. Although some studies have reported positive user experience or education-related outcomes (38, 40, 41), the overall evidence is still dominated by methodological and descriptive outcomes, making it difficult to support robust inferences about the effects of knowledge graphs in nursing practice. This evidence distribution characteristic suggests that the development of knowledge graphs in nursing at this stage is more a form of technological exploration and scenario testing rather than a mature evidence-based application tool. This phenomenon of technology leading and evidence lagging also provides important clues for the key methods of subsequent research.

### 4.3 Structural fractures from knowledge construction to application translation

Evidence maturity analysis showed that studies in the Construction and System Performance Evaluation Phases accounted for the highest proportion, but most of these studies only stayed at the level of ontology design, entity-relationship extraction, and system architecture verification, with outcome indicators mainly being knowledge graph scale or prediction model performance parameters. There is a lack of clinical or behavioral outcomes directly related to nursing practice, which to a certain extent limits the in-depth translation of knowledge graphs to nursing practice scenarios. In addition, some studies completed verification in laboratory or offline environments, but there is an irreconcilable tension between the controllability of laboratory environments and the complexity of clinical environments. A scoping review pointed out that prediction models perform excellently in offline environment simulations but show a significant decline in performance in clinical environments (44). This tension is not a flaw in study design but a common challenge in the technology translation process. Conducting prospective verification studies and incorporating more contextual variables into model design may be feasible paths to bridge this tension.

### 4.4 Evidence characteristics and limitations of usability evaluation and preliminary application studies

In the Usability Evaluation and Preliminary Application Phases, studies began to embed in specific nursing scenarios and focus on indicators such as user experience, learning support, or system participation. Studies have shown (16, 29, 34, 35) that users gave positive evaluations of knowledge visualization and interface usability, and also put forward specific improvement suggestions such as supplementary analysis and optimized navigation, providing a clear direction for subsequent system iteration. However, due to the lack of controlled comparison and insufficient standardization of outcome indicators, existing evidence still cannot distinguish the effect of knowledge graphs themselves from the influence of confounding factors such as teaching models and novelty of use. For example, it is impossible to determine from the existing design whether the high willingness of “99% of students to continue using” (39) is entirely attributed to knowledge graphs or partially to the freshness of participating in the research. This limitation in the evidence level means that current research mostly reflects feasibility and acceptability rather than real effect verification. In addition, the strength of the correlation between subjective satisfaction and actual behavior is questionable. For example, in a study on a nostalgic photo album program, 92% of users considered it easy to use, but only 69% hoped to use it frequently (29). A systematic review in the digital health field also pointed out that high satisfaction (>80%) coexists with low actual usage rate and poor health improvement (45). This evidence gap of “users report positive feedback but actual behavior is unvalidated” leads to a lack of effect estimation basis for subsequent phases. Objective behavioral indicators (e.g., actual login frequency, depth of function use, number of knowledge queries) should be tracked synchronously in usability evaluation to establish correlational evidence between subjective satisfaction and objective behavior, providing a basis for sample size estimation for subsequent effect verification.

### 4.5 Concentration of studies in the Application Phase

The number of studies that have truly entered the Application Phase with controlled designs is limited, and all are concentrated in nursing education scenarios. These studies introduced controlled comparison designs for the first time and reported objective outcome indicators such as course scores and independent learning ability. Studies by Bao et al. (40) and Song et al. (41) showed that students using knowledge graphs had better course scores than the control group (*p* < 0.001), providing preliminary evidence for the effectiveness of knowledge graphs in nursing education scenarios. However, research on clinical nursing decision support and disease management has stagnated at the Usability Evaluation or System Performance Evaluation Phase, with no verification in the Application Phase. This field differentiation of “easy in education, difficult in clinical practice” is not caused by technical differences but by the structural mechanism of publication bias: nursing education scenarios have high controllability, low ethical thresholds, and easily measurable outcomes, while clinical scenarios face difficulties in data standardization, complex system integration, and patient safety concerns (46). This may lead to the evidence system tilting toward low-risk scenarios, while evidence accumulation in high-value clinical application scenarios is relatively lagging. Accumulating evidence from scenarios such as nursing training, quality monitoring, and outpatient education, and reducing clinical access thresholds by using small-sample before-and-after self-control and single-module verification, and gradually penetrating into high-risk clinical scenarios can gradually bridge this gap.

### 4.6 Literature gaps and future research opportunities

The application of knowledge graphs in nursing has shown potential in multiple scenarios and forms. However, the overall research is still in the stage of construction and preliminary exploration, with a serious shortage of clinical empirical evidence and the need to strengthen technical maturity and application depth. Similar issues have also been mentioned in a systematic review of knowledge graphs by Gui et al. (47). Future research must make joint efforts in the following key directions to promote its systematic improvement and substantive application:

1. Promote the migration of evidence to clinical scenarios: The current evidence system shows a structural imbalance of “boom in education, slump in clinical practice.” Future research should prioritize applied research on knowledge graphs in scenarios such as clinical nursing decision support, chronic disease management, and high-risk patient monitoring, and explore low-risk entry strategies, starting with providing reference suggestions to reduce patient safety concerns.
2. Improve the strength of causal inference in research design: RCTs are completely missing in the current evidence chain, with non-randomized grouping, historical control, and single-group design dominating, making it difficult to establish a causal relationship between knowledge graphs and outcome improvement. In nursing education scenarios, the existing cluster randomization design should be optimized (e.g., adopting stratified randomization and controlling intra-group correlation) to solve the current problem of “having a design but insufficient efficiency.” In clinical scenarios, stepped wedge randomized controlled trials can be adopted to promote popularization gradually while maintaining control groups.
3. Promote data standardization and cross-institutional transfer: Knowledge graph construction relies on academic literatures and textbooks, with insufficient integration of real clinical data and a lack of unified terminology standards, leading to a disconnect between knowledge graphs and bedside practice and difficulties in cross-institutional reuse. The lack of unified metadata standards and semantic mapping specifications limits the semantic interoperability across institutions and systems, which restricts the universality and sharing capacity of knowledge graphs (48). Future research should establish core nursing ontologies and metadata standards, promote the localized mapping of international nursing terminology (e.g., ICNP) and clinical terminology, explore standardized extraction of unstructured data such as electronic medical records and nursing documentation and automatic knowledge graph population technology, and conduct multicenter validation studies to test the applicability of knowledge graphs in different healthcare systems and cultural backgrounds.

## 5 Limitations

This review has several limitations. First, despite the systematic search, the included literatures are dominated by Chinese studies. This geographical concentration is not merely a demographic observation—it carries substantive implications for how the evidence base is perceived. The dominance of Chinese studies likely overestimates the feasibility and effectiveness of knowledge graphs in nursing education, as 8 of the 9 studies in the usability and Application Phases that reported positive educational outcomes were conducted in China. Conversely, it may underrepresent clinical effectiveness research conducted in Western healthcare systems, where higher regulatory barriers, complex EHR integration, and different data governance models may slow down or limit KG implementation. This imbalance creates a risk of “evidence distortion”: readers may conclude that knowledge graphs are “proven to work” in nursing, when in fact the proof is concentrated in a single country and a single scenario (education). Second, due to the significant heterogeneity of the original studies in application scenarios, technical methods, and evaluation indicators, this study was unable to conduct in-depth quantitative comparison or analysis of the pros and cons of technical routes, and mostly conducted descriptive integration and classification. The relevant conclusions should be regarded as exploratory rather than causal evidence. Finally, the stratification of evidence maturity aims to describe the research Application Phase rather than a formal quality evaluation, and may not fully reflect the inherent methodological differences of individual studies. Future research can conduct further systematic reviews or quantitative comprehensive analysis on the basis of gradual evidence accumulation.

## 6 Conclusion

This study indicates that research on knowledge graphs in the nursing field is still in the exploratory stage overall. Although relevant research has been continuously expanded in application scenarios and technical forms, existing evidence is mainly concentrated on technical construction and usability evaluation, and the number of studies with controlled designs and objective outcome reporting is limited. There is a lack of objective evidence to support whether knowledge graphs truly change nursing behaviors, improve nursing quality, and enhance patient health outcomes. Future research urgently needs to shift from technology-driven to evidence-driven, focus on the application effects in real nursing contexts, and construct an evaluation framework centered on nursing-sensitive outcomes. By improving the rigor of research design and the clinical relevance of outcome indicators, knowledge graphs are expected to play a more evidence-based supporting role in nursing education and practice.
