# Supplementary material for: Application scope of knowledge graphs in nursing: a scoping review
Source: Front Public Health. 2026 Apr 15;14:1763063. doi: 10.3389/fpubh.2026.1763063 (PMC13127119; doi:10.3389/fpubh.2026.1763063)
Supplement: Supplementary file 2 [file Supplementary_file_1.docx]

**Supplementary File 1**

Table 1:

Search strategy table.

| Database |  | Search Strategy | Number of  Hits |
| --- | --- | --- | --- |
| 知网(CNKI) | #1 | 知识图谱 + 语义网络 + 语义网 + 本体 + 图谱构建 + 知识表示 + 知识抽取 + 图数据库 + Neo4j | 154,696 |
|  | #2 | 护理学 + 护理 + 护理教育 + 护理科研 + 临床护理 + 护理信息化 + 智慧护理 + 患者教育 + 健康管理 | 1,588,377 |
|  | #3 | #1 AND #2 | 731 |
| 万方(WanFang Data) | #1 | 知识图谱 OR 语义网络 OR 语义网 OR 本体 OR 图谱构建 OR 知识表示 OR 知识抽取 OR 图数据库 OR Neo4j | 263,910 |
|  | #2 | 护理学 OR 护理 OR 护理教育 OR 护理科研 OR 临床护理 OR 护理信息化 OR 智慧护理 OR 患者教育 OR 健康管理 | 2,595,904 |
|  | #3 | #1 AND #2 | 2,002 |
| 维普(VIP) | #1 | 知识图谱 OR 语义网络 OR 语义网 OR 本体 OR 图谱构建 OR 知识表示 OR 知识抽取 OR 图数据库 OR Neo4j | 71,245 |
|  | #2 | 护理学 OR 护理 OR 护理教育 OR 护理科研 OR 临床护理 OR 护理信息化 OR 智慧护理 OR 患者教育 OR 健康管理 | 1,267,621 |
|  | #3 | #1 AND #2 | 271 |
| 中国生物医学文献服务系统  (SinoMed) | #1 | ( "知识图谱"[关键词:智能] OR "语义网络"[关键词:智能] OR "语义网"[关键词:智能] OR "本体"[关键词:智能] OR "图谱构建"[关键词:智能] OR "知识表示"[关键词:智能] OR "知识抽取"[关键词:智能] OR "图数据库"[关键词:智能] OR "Neo4j"[关键词:智能]) | 4,301 |
|  | #2 | ( "护理学"[关键词:智能] OR "护理"[关键词:智能] OR "护理教育"[关键词:智能] OR "护理科研"[关键词:智能] OR "临床护理"[关键词:智能] OR "护理信息化"[关键词:智能] OR "智慧护理"[关键词:智能] OR "患者教育"[关键词:智能] OR "健康管理"[关键词:智能]) | 299,857 |
|  | #3 | #1 AND #2 | 60 |
| PubMed | #1 | "knowledge graphs"[Title/Abstract] OR "semantic network*"[Title/Abstract] OR "ontology"[Title/Abstract] OR "knowledge representation"[Title/Abstract] OR "knowledge extraction"[Title/Abstract] OR "graph database"[Title/Abstract] OR "Neo4j"[Title/Abstract] | 52,360 |
|  | #2 | "Nursing"[MeSH Terms] | 271,599 |
|  | #3 | "Nursing Care"[MeSH Terms] | 145,949 |
|  | #4 | "nursing education"[Title/Abstract] OR "nursing research"[Title/Abstract] OR "clinical nursing"[Title/Abstract] OR "patient education"[Title/Abstract] | 63,576 |
|  | #5 | #2 OR #3 OR #4 | 357,567 |
|  | #6 | #1 AND #5 | 245 |
| Embase | #1 | 'knowledge graphs':ti,ab,kw OR 'semantic network*':ti,ab,kw OR ontology:ti,ab,kw OR 'knowledge representation':ti,ab,kw OR 'knowledge extraction':ti,ab,kw OR 'graph database':ti,ab,kw OR neo4j:ti,ab,kw | 62,662 |
|  | #2 | nursing:ti,ab,kw OR 'nursing care':ti,ab,kw OR 'nursing education':ti,ab,kw OR 'nursing research':ti,ab,kw OR 'clinical nursing':ti,ab,kw OR 'patient education':ti,ab,kw | 458,828 |
|  | #3 | #1 AND #2 | 263 |
| Web of science | #1 | (TS=("knowledge graphs" OR "semantic network*" OR "ontology" OR "knowledge representation" OR "knowledge extraction" OR "graph database" OR neo4j)) | 347,883 |
|  | #2 | (TS=("nursing" OR "nursing care" OR "nursing education" OR "nursing research" OR "clinical nursing" OR patient education)) | 415,386 |
|  | #3 | #1 AND #2 | 1,729 |
| CINAHL | #1 | SU (Knowledge Graphs OR semantic network* OR ontology OR knowledge representation OR knowledge extraction OR graph database OR Neo4j) | 44,027 |
|  | #2 | SU (Nursing OR Nursing Care OR nursing education OR nursing research OR clinical nursing OR patient education) | 2,346,220 |
|  | #3 | #1 AND #2 | 322 |
| Cochrane Library | #1 | ("knowledge graphs" OR "semantic network*" OR "ontology" OR "knowledge representation" OR "knowledge extraction" OR "graph database" OR neo4j):ti,ab | 465 |
|  | #2 | MeSH descriptor: [Nursing] explode all trees | 4,450 |
|  | #3 | MeSH descriptor: [Nursing Care] explode all trees | 2,422 |
|  | #4 | ("nursing education" OR "nursing research" OR "clinical nursing" OR patient education):ti,ab | 104,262 |
|  | #5 | #2 OR #3 OR #4 | 110.567 |
|  | #6 | #1 AND #5 | 131 |
